# Evidence for thermosensitivity of the cotton (*Gossypium hirsutum* L.) immature fiber (*im*) mutant via hypersensitive stomatal activity

**DOI:** 10.1371/journal.pone.0259562

**Published:** 2021-12-13

**Authors:** Hee Jin Kim, Naohiro Kato, Ruth Ndathe, Gregory N. Thyssen, Don C. Jones, Harish H. Ratnayaka

**Affiliations:** 1 USDA-ARS, Southern Regional Research Center, Cotton Fiber Bioscience Research Unit, New Orleans, LA, United States of America; 2 Department of Biological Sciences, Louisiana State University, Baton Rouge, LA, United States of America; 3 Cotton Incorporated, Cary, NC, United States of America; 4 Department of Biology, Xavier University of Louisiana, New Orleans, LA, United States of America; University of Tasmania, AUSTRALIA

## Abstract

Thickness of cotton fiber, referred to as fiber maturity, is a key determinant of fiber quality, lint yield, and textile performance. The cotton immature fiber (*im*) mutant has been used to study fiber maturity since its fiber is thinner than the wild type near isogeneic line (NIL), Texas Marker-1 (TM-1). The *im* phenotype is caused by a single recessive mutation of a pentatricopeptide repeat (*PPR*) gene that reduces the activity of mitochondrial complex I and up-regulates stress responsive genes. However, the mechanisms altering the stress responses in *im* mutant are not well understood. Thus, we characterized growth and gas exchange in *im* and TM-1 under no stress and also investigated their stress responses by comparing gas exchange and transcriptomic profiles under high temperature. Phenotypic differences were detected between the NILs in non-fiber tissues although less pronounced than the variation in fibers. At near optimum temperature (28±3°C), *im* maintained the same photosynthetic performance as TM-1 by means of greater stomatal conductance. In contrast, under high temperature stress (>34°C), *im* leaves reduced photosynthesis by decreasing the stomatal conductance disproportionately more than TM-1. Transcriptomic analyses showed that the genes involved in heat stress responses were differentially expressed between the NIL leaves. These results indicate that the *im* mutant previously reported to have low activity of mitochondrial complex I displays increased thermosensitivity by impacting stomatal conductance. They also support a notion that mitochondrial complex I activity is required for maintenance of optimal photosynthetic performance and acclimation of plants to high temperature stress. These findings may be useful in the future efforts to understand how physiological mechanisms play a role in determining cotton fiber maturity and may influence stress responses in other crops.

## Introduction

Cotton (*Gossypium* sp.) is the world’s most important natural fiber [1]. Upland cotton (*Gossypium hirsutum* L.) is an allotetraploid and accounts for 97% of the global cotton production [2]. Botanically, cotton fibers are dry trichome cells that normally have a thick secondary cell wall (SCW) consisting almost entirely of cellulose [3, 4]. During the development of cotton fiber, its cell wall thickens as the β-1,4-glucan chains form the highly organized cellulose microfibrils (CMFs) structure and pack in the SCW [5, 6]. Fiber maturity representing the degree of thickening in the fiber cell wall is a crucial determinant of the yield, quality and commercial value of cotton [7, 8]. The degree of fiber wall thickness (θ) is theoretically calculated using an equation, θ = 4πA/P^2^ where A and P are average cell wall area and perimeter respectively determined from microscopic images of cross-sectioned fibers [9, 10]. Due to difficulties in measuring θ values, textile industry uses the micronaire (MIC) values determined by measuring the air-flow resistance of a certain weight of cotton fibers [7, 8] as an estimate of the fiber maturity of most cotton [11–19].

A cotton mutant producing immature fiber was discovered in the early 1970s from an upland cotton variety Acala 4–42 [20]. By back-crossing this immature fiber (*im*) mutant multiple times with a standard Texas Marker-1 (TM-1), a pair of near isogenic lines (NILs) differing in fiber maturity were generated in 1990 [11]. Though there were significant reductions in both θ and MIC values of *im* fibers compared with TM-1, their non-fiber phenotypes have been found so far to be similar [11, 14, 15]. Thus, these NIL plants have been widely used as a model system for studying maturity and SCW biosynthesis during cotton fiber development [21–23]. Multiple genetic analyses commonly identified that the *im* phenotype is controlled by a single recessive *im* gene located on the chromosome A03 [12, 14, 15]. Through a mapping-by-sequencing, we previously identified that a 22-bp deletion mutation of a gene (*imPPR*, Ghir_A03G006650) encoding pentatricopeptide repeat (PPR) motifs was completely linked to the aberrant *im* fiber phenotype [24]. Transcriptomic profiles of the NIL fibers suggested that the *im* phenotype may be associated with deregulation of the genes involved in two biological processes, including mitochondrial cellular respiration and stress responses [13]. The effects of the *im* mutation on the mitochondrial respiration has been verified by a recent functional analysis of *im* plants showing that the imPPR protein (also named *Gh*ImA) is responsible for splicing mRNA encoding *NADH dehydrogenase subunit 7* (*nad7*) of the mitochondrial complex I [25]. However, mechanisms of the stress responses in *im* mutant are not known mainly due to the difficulty to perform biological analyses with cotton fibers mostly composed of SCW cellulose [5, 6].

Plant stress responses are often measured from the leaf tissue since leaf photosynthesis is highly sensitive to sudden changes in environmental conditions and serves as a sensor of abiotic stress [26–29]. Abiotic stress reduces the leaf photosynthetic performance by inducing stomatal closure [27, 28, 30]. Effects of abiotic stress on stomatal aperture regulation through abscisic acid (ABA) signaling pathway have been well characterized [31, 32]. Furthermore, abiotic stress often inhibits light absorption, energy distribution and electron transport that are critical for the photosystem II (PSII) activity and carbon assimilation [30, 33–35]. Photosynthetic performance in cotton leaves is also affected by the environmental conditions and ultimately influences the development of fiber cell wall composed of the homopolymer of glucose, cellulose [6] and growth characteristics. Thus, we first studied the leaf mass per unit area (LMA) and biomass of different organs to understand if growth characteristics are affected in the *im* plants compared with TM-1. We then used photosynthetic physiology for testing if there are differences in responses to high temperature stress between the NILs. Furthermore, comparative transcriptomic profiles were used to verify the physiological responses of the NIL plants to high temperature stress. The findings of these investigations provide novel insight into how growth characteristics and stress tolerance have been affected together with the fiber maturity in the *im* plants compared with the TM-1.

## Materials and methods

### Plant material

**Fig 1:**
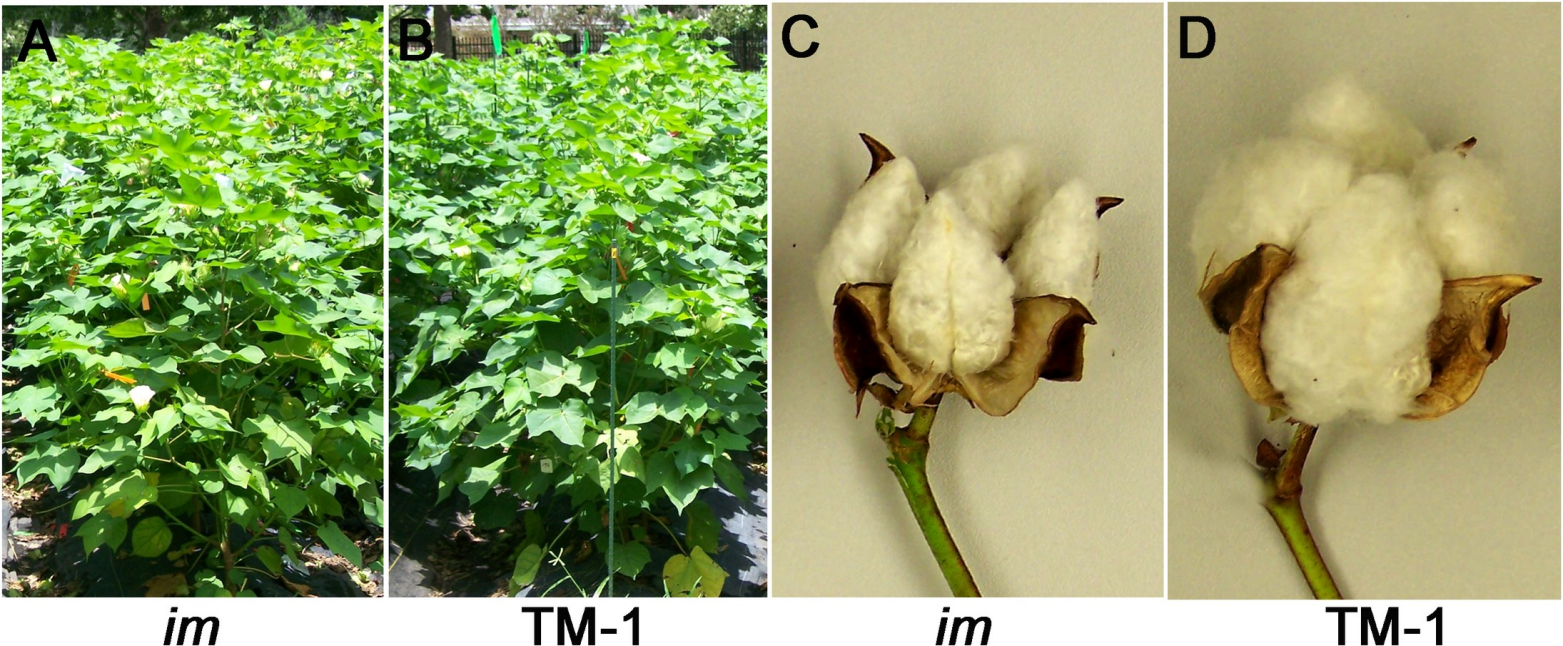
Phenotypic comparisons of the two upland cotton lines, immature fiber (*im*) mutant and its isogenic wild type, Texas Marker-1 (TM-1). A. A field row of *im* plants. B. A field row of TM-1 plants. The length of a green garden stake used as a scale marker is 120 cm. C. The non-fluffy phenotype of the *im* boll. D. The fluffy phenotype of the TM-1 boll.

Field research was performed to collect cotton materials according to the policy and practices of USDA-ARS. The cottonseeds of the two NILs, *im* and TM-1, were provided by Dr. Russell J. Kohel of USDA-ARS-SPARC who originally developed the NILs [11]. The NIL plants (Fig 1) were grown in the fields (USDA-ARS, College Station, TX in 2007, and USDA-ARS, New Orleans in 2014 and 2015), greenhouses and a growth chamber (New Orleans), in fully randomized designs and treated equivalently within each environment during the growth period. Field-grown plants were used for the comparative analyses of fiber and LMA as well as physiological investigations including the effect of high temperature treatments applied locally on the leaves. Greenhouse- and growth chamber-grown plants were used for leaf RNA-seq analyses after the heat treatments of the whole plants. The College Station field was composed of high percentage of clay, and the New Orleans field was Aquent dredged over alluvium in an elevated location to provide adequate drainage. In each field, 50 plants of each NIL were grown with standard cultural practices. For the whole plant heat treatments, NILs (two plants) were grown in 14L pots in a greenhouse and transferred to the growth chamber (Percival Intellus Environmental Controller, Perry, IA) 12 weeks after planting. Growth chamber temperature was set at 28°C with 16h day light (300 μmol m^-2^ s^-1^) to acclimate the plants to near optimum temperature for 3 days and increased to 35°C for three days for the heat treatment. Leaf samples from 2 plants of each NIL were taken for RNA-seq investigations. The soil used in all pots was Metro-Mix 360 (Hummert International, Earth City, MO) top-dressed with Osmocote (15:9:12) fertilizer.

### Measurements of fiber properties and leaf mass per unit area of NILs

The fully developed fibers were ginned using a laboratory roller gin. All fibers were pre-equilibrated at 65% humidity and 21°C air temperature for 48 hours before the measurements of fiber property. Fiber properties including MIC, elongation (ELO), length (UHML), and uniformity (UI) of NILs were measured by a high volume instrument (HVI) (USTER Technologies Inc., Charlotte, NC). Fully expanded leaves were harvested at the position of 18^th^ node from ten NIL plants grown in the 2015 field season with high temperatures (>34°C) in most afternoons during the vegetative and reproductive development. Three discs were collected from each leaf with a cork borer of known diameter. To reduce sample variation, leaf midrib and prominent secondary veins were avoided. The discs were dried to a constant mass at 60°C in an oven and weighed. Average LMA values (g/m^2^) were calculated from 30 dried discs.

### Biomass of different plant organs of NILs

For determining the biomass in different plant organs, the NILs were grown in 14L pots (six replicates) in the greenhouse. Roots, stems, leaves, petioles and reproductive organs (square, flower, boll, bract and peduncle combined) were harvested separately with clippers at early flowering stage (9 weeks after planting). Biomass was measured by drying each tissue type in an oven at 65°C until constant weight, and weighing with a microbalance.

### Calculation of heat unit from the field grown NILs

Daily temperature records of the cotton fields for the year 2007, 2014, and 2015 were downloaded from a weather history web site (http://api.wunderground.com/history). Heat unit or growing degree days (GDD) was calculated from the equation, GDD = $\sum [\frac{Tmax+Tmin}{2}-Tt]$ where *Tmax* and *Tmin* are the daily maximum and minimum temperatures respectively, and *Tt* is the threshold temperature (60°F or 15.6°C) required for cotton growth and development [36, 37]

### Gas exchange and photosystem II measurements with or without high leaf temperature treatment in field

Field-grown, sixteen-week-old NIL plants were used for photosynthetic measurements.

Net photosynthesis rate (*P*_net_) and other gas exchange characteristics were measured using LI 6400XT (LI-COR Inc. Lincoln, NE, USA) using the leaves at the 18^th^ node (1^st^ position) in 6 plants each of *im* or TM-1 grown together. Measurements were taken at internal photon flux 1500 µmol m^-2^ s^-1^, flow rate 200 µmol s^-1^, internal CO_2_ concentration 400 µmol mol^-1^, block temperature 32°C and sample RH 60±2%. Each measurement was recorded only after *P*_net_ and stomatal conductance (*g*_s_) stabilized as observed on the display of the LI 6400XT console. The same field-grown plants were used to measure the gas exchange from the same leaves at leaf temperatures of ~30, ~35 or ~42°C, instead of block temperature, maintained using LI 6400XT. All other LI 6400XT settings were the same as used earlier. The *im* and TM-1 plants were alternatively measured at each leaf temperature starting at ~30 and finishing at 42°C. Rapid light response was measured to understand if the similar photosynthetic performance between NILs seen at the 1500 µmol m^-2^ s^-1^ light level would continue in a changing light environment too. The same leaf samples used for the above-mentioned gas exchange measurements were subjected to photon flux levels of 2500, 2250, 1750, 1500, 1250, 1000, 750, 500, 250, 100, 50 and 0 µmol m^-2^ s^-1^ working from high to low light intensity using the LI 6400XT autoprogram with minimum and maximum wait times of 3 and 4 min, respectively, per measurement.

Photosystem II measurements were performed from the same leaves used for the above gas exchange measurements with Hansatech FMS-2 modulated fluorometer (FMS 2, Hansatech Instruments Ltd., Norfolk, UK) after dark-adapting for at least 60 min with the clips provided by the manufacturer. Maximum photosystem II efficiency (*F*_v_/*F*_m_) and related fluorescence parameters were recorded concurrently using the script editor settings of gain 80, modulation 3, FvFm 2.5 90 0.7, actinic light 20, wait 80 and PS2 2.5 90 0.7 of the FMS 2.

### RNA-seq analyses of heat-stressed whole plants in growth chamber

The NIL plants that were grown in 14L pots in the greenhouse as mentioned in the plant material section were used for RNA-seq analyses. Whole NIL plants that were previously acclimated to 28°C were incubated at 35°C for 3 days as the heat treatment. Total RNA was extracted from these heat-treated plants using the Sigma Spectrum™ Plant Total RNA Kit (Sigma-Aldrich, St. Louis, MO) with DNase1 digestion according to the manufacturer’s protocol. The quality and quantity of total RNA were determined using a NanoDrop 2000 spectrophotometer (NanoDrop Technologies Inc., Wilmington, DE) and an Agilent Bioanalyzer 2100 (Agilent Technologies Inc., Santa Clara, CA). RNA-seq analyses were performed with the RNA samples of two biological replications. Paired 150-bp Illumina RNA-seq reads were aligned to a reference *Gossypium hirsutum* cv TM-1 genome [38] and to the *G*. *hirsutum* plastid and mitochondrial genomes (DQ345959 and JX065074) simultaneously using HISAT2 software [39]. Reads mapping to annotated protein sequences were counted using BEDTools software [40], and the counts per gene were normalized by the reads per kilobase of coding sequence per millions of mapped reads (RPKM). Statistical analyses were performed with false discovery rate (FDR) < 0.05 by at least 2-fold differences of expression levels for identifying Differentially Expressed Genes (DEGs). The gene ontology (GO) enrichment analysis of the heat treated NIL leaves was performed using the agriGO Singular Enrichment Analysis [41]. For GO enrichment, the *p*-value cutoff for significance was 0.05. For identifying commonly regulated genes from the NIL leaves (accession number PRJNA610034) and developing NIL fibers at 28 DPA [24], the cotton DEGs were compared with *Arabidopsis* genome sequences of The *Arabidopsis* Information Resource version 10 (TAIR 10) [42] and annotated based on the function of the *Arabidopsis* genes that were the best hit by BLAST search [43].

### Statistical analyses

Data analyses and tests for statistical significances of the treatments for each response variable related to photosynthesis were performed using general linear model on SPSS version 19.0.0.1 (International Business Machines, 2010). Pairwise comparisons after significant ANOVA were performed for with Tukey’s HSD. Phenotypic and statistical analyses for calculating correlation coefficient (*r*) and R-squared (*R*^*2*^) were performed using t-test and two-way ANOVA with Prism version 7.1 software (Graph-Pad Software, Inc., San Diego, CA) and excel program. The *p* value cutoff for significance was 0.05.

## Results

### Phenotypic differences of the field-grown NIL fibers

Fiber properties of the NIL plants grown under field conditions were measured with an automated HVI instrument. As shown in Table 1, fiber MIC of the *im* plants (3.17) was 24.2% (*p* < 0.0001) lower than the TM-1 plants (4.18). In contrast, the other fiber properties such as elongation, length, and uniformity showed insignificant differences between the NILs (Table 1).

**Table 1:** Fiber properties and leaf specific mass of the field-grown *im* and TM-1 plants.

Tissue type	Properties	*im*			TM-1			Ratio (%) *im*/TM-1
		Mean	SD	N	Mean	SD	N	
**Fiber**	**MIC**	3.17	0.09	5	4.18	0.25	5	75.80****
	**ELO (%)**	4.54	0.30	5	4.84	0.29	5	93.80 (ns)
	**UHML (mm)**	31.29	0.66	5	32.24	0.76	5	97.05 (ns)
	**UI (%)**	85.42	0.63	5	86.16	0.95	5	99.14 (ns)
**Leaf**	**LMA (g/m** ^**2**^ **)**	78.3	1.6	30	86.5	1.1	30	90.60***

MIC, micronaire; ELO, elongation; UHML, upper half mean length; UI, uniformity; LMA, leaf mass per unit area;

***, *p* < 0.001;

****, *p* < 0.0001; ns, not significant.

**Fig 2:**
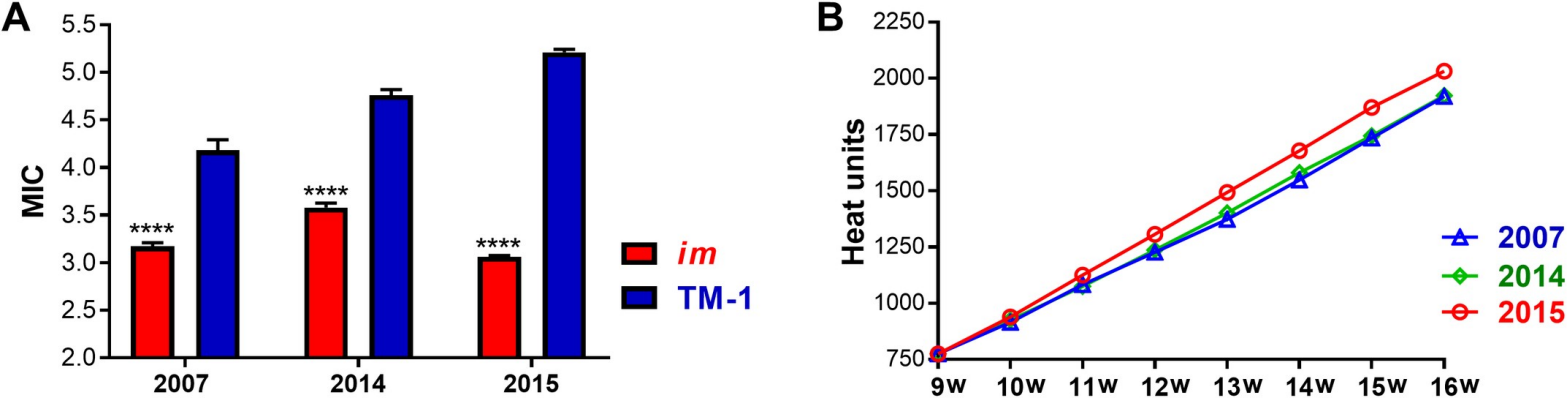
The fiber MIC of the NIL plants and the heat units during their active fiber development in the three field seasons. A. Comparisons of MIC values between the *im* and TM-1 plants. Statistical significance at *p* < 0.0001 = ****. Error bar represents standard error of the mean. B. Heat units of three field seasons during active fiber development. Heat unit was determined by calculating growing degree days (GDD).

The MIC values of the field-grown *im* fibers (3.17, 3.68, and 3.06) of all three seasons (Year 2007, 2014, and 2015) were significantly lower than the MIC values of the corresponding TM-1 fibers (4.18, 4.76, and 5.21) despite fluctuations of the MIC values (Fig 2A). Among the three field seasons, the lowest MIC value of the *im* fibers (3.06) and the highest MIC value of the TM-1 fibers (5.21) were found in 2015. Thus, the MIC reduction of the *im* fibers compared with the TM-1 fibers was much more severe (41.3%) in 2015 than in both 2007 (24.2%) and 2014 (25.6%). In all three seasons, seeds were planted similarly in early May. The first flower commonly formed in early July (9^th^ week after planting), and the entire fiber development required 45~50 days. Mature fibers were collected in early September (17^th^ week after planting) for each year. The heat map of the average weekly maximum temperature during the period of active fiber development of July and August (9^th^ to 16^th^ week) of the three seasons showed that the plants in 2015 experienced a longer period of high temperature, starting mid-July through mid-August, compared with the plants in other seasons (S1 Fig). Unlike the high temperature stress that prevailed in the afternoon, cold temperature stress (<23°C) [44, 45] during night times did not exist in any of the three seasons in our field (S2 Fig). The daily weather records of the field showed that the plants experienced high temperature stress (>34°C) [46, 47] from 11 A.M. to 6 P.M during the period of active fiber development in 2015 (S3 Fig). Heat unit comparisons showed that the 2015 season which produced the lowest MIC ratio in *im* fibers (Fig 2A) among the three seasons had the greatest GDD during the period of the fiber development (Fig 2B).

### Significant differences in, leaf mass per unit area (LMA) and biomass between the NILs

We tested if the field-grown NILs that experienced the extended period of high temperature and had the distinctive MIC variation in 2015 showed differences in leaf phenotype. Leaf mass per unit area (LMA) was measured for leaf phenotypic comparison as MIC was used for fiber maturity. Consistent with the significantly lower MIC of *im* fibers (Fig 2A), their LMA (78.3 ± 1.6 g/m^2^) at the same node and branch position also was significantly lower (9.4%, *p*< 0.001) than TM-1 (86.5 ± 1.1 g/m^2^) compared with TM-1 as shown in Table 1.

**Fig 3:**
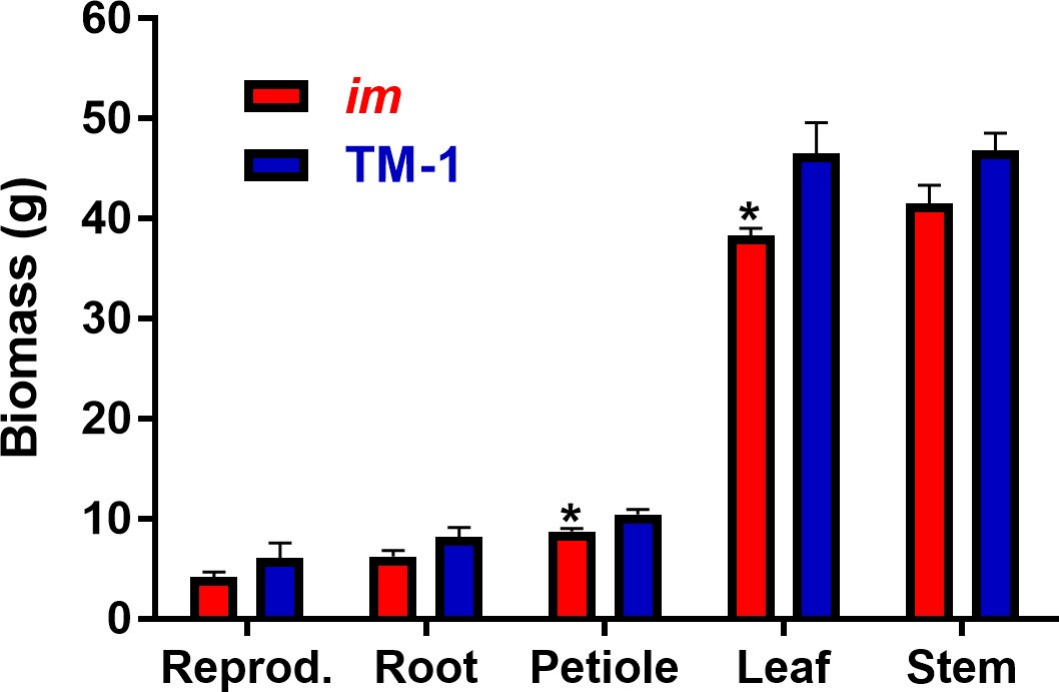
Biomass of different organs of the NIL plants grown in greenhouse. Each of the *im* or TM-1 plants was grown in a 14L pot with six replications. Average biomass of the whole plant as well as each type of organs including reproductive (bract, square, flower, boll and peduncle combined), root, petiole, leaf, and stem of the *im* plants were compared with TM-1. Two-way ANOVA showed a significant difference (*p* < 0.0001) in the total biomass between NIL plants. Error bar represents standard error of the mean. Biomass difference of each organ between the NILs was tested with t-tests and * above the bar indicates statistical significance (*p* < 0.05).

We also used six each of NIL plants grown in 14L pots in a greenhouse under ambient temperature to compare the biomass of reproductive (bract, square, flower, boll and peduncle combined), root, petiole, leaf and stem organs separately. Two-way ANOVA showed that the total biomass of the *im* plants (98.95g ± 3.94g) was significantly (*p* < 0.0001) lower in *im* than the TM-1 (118.05g ± 7.68g) plants. Although each individual type of organs also had lower biomass in *im* only the leaf and petiole showed significant differences compared with TM-1 (Fig 3).

### Similar net photosynthesis with different stomatal conductance of the field-grown NIL leaves at optimum temperatures

**Fig 4:**
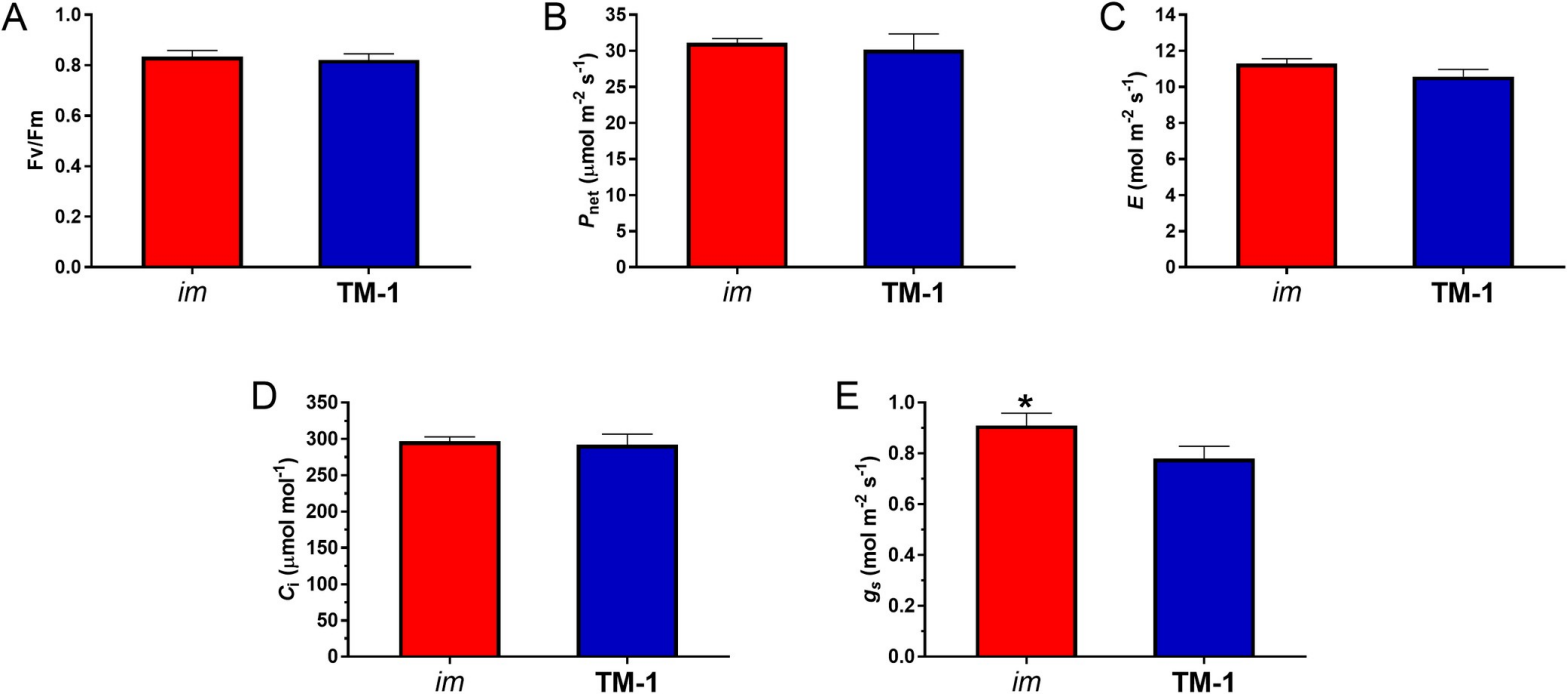
Photosynthetic variables of the field-grown *im* and TM-1 plants at an optimum temperature. Photosynthetic variables including (A) *F*_v_/*F*_m_, maximum quantum yield of PSII photochemistry, (B) *P*_net_, net photosynthesis, (C) *E*, transpiration rate (D) *C*_i_, intercellular CO_2_ concentration, and (E) *g*_s_, stomatal conductance were measured from the leaf at the 18^th^ node of the NILs at 31°C, within the range of the optimum temperature (28 ± 3°C). Error bar represents standard error of the mean. *, *p* < 0.05.

Leaf PSII efficiency and gas exchange variables were measured to investigate light-dependent and -independent processes of photosynthesis in the field-grown NIL leaves at the 18^th^ node (top canopy exposed to full sun light 1,400~1,600 µmol m^-2^s^-1^) in a mid-morning of August 2015 when the temperatures of the *im* (30.95°C) and TM-1 (31.10°C) leaves were close to the optimum (28 ± 3°C) for the field-grown upland cotton with irrigation [46]. The maximum PSII efficiency (*F*_v_/*F*_m_) showed no significant difference between the *im* and TM-1 plants (Fig 4A). The gas exchange measurements showed similar (*p =* 0.18) net photosynthesis (*P*_net_) between *im* (31.13 µmol m^-2^s^-1^) and TM-1 (30.16 µmol m^-2^s^-1^) (Fig 4B). In addition, transpiration rate (*E*) and intercellular CO_2_ concentration (*C*_i_) were also similar between the NIL leaves (Fig 4C and 4D). However, stomatal conductance (*g*_s_) of the *im* leaves (0.91 mol m^-2^s^-1^) was significantly greater (17%) than TM-1 (0.78 mol m^-2^s^-1^) as shown in Fig 4E.

### Persistent similarity in photosynthesis between the NIL leaves treated with variable light conditions at a constant temperature

**Fig 5:**
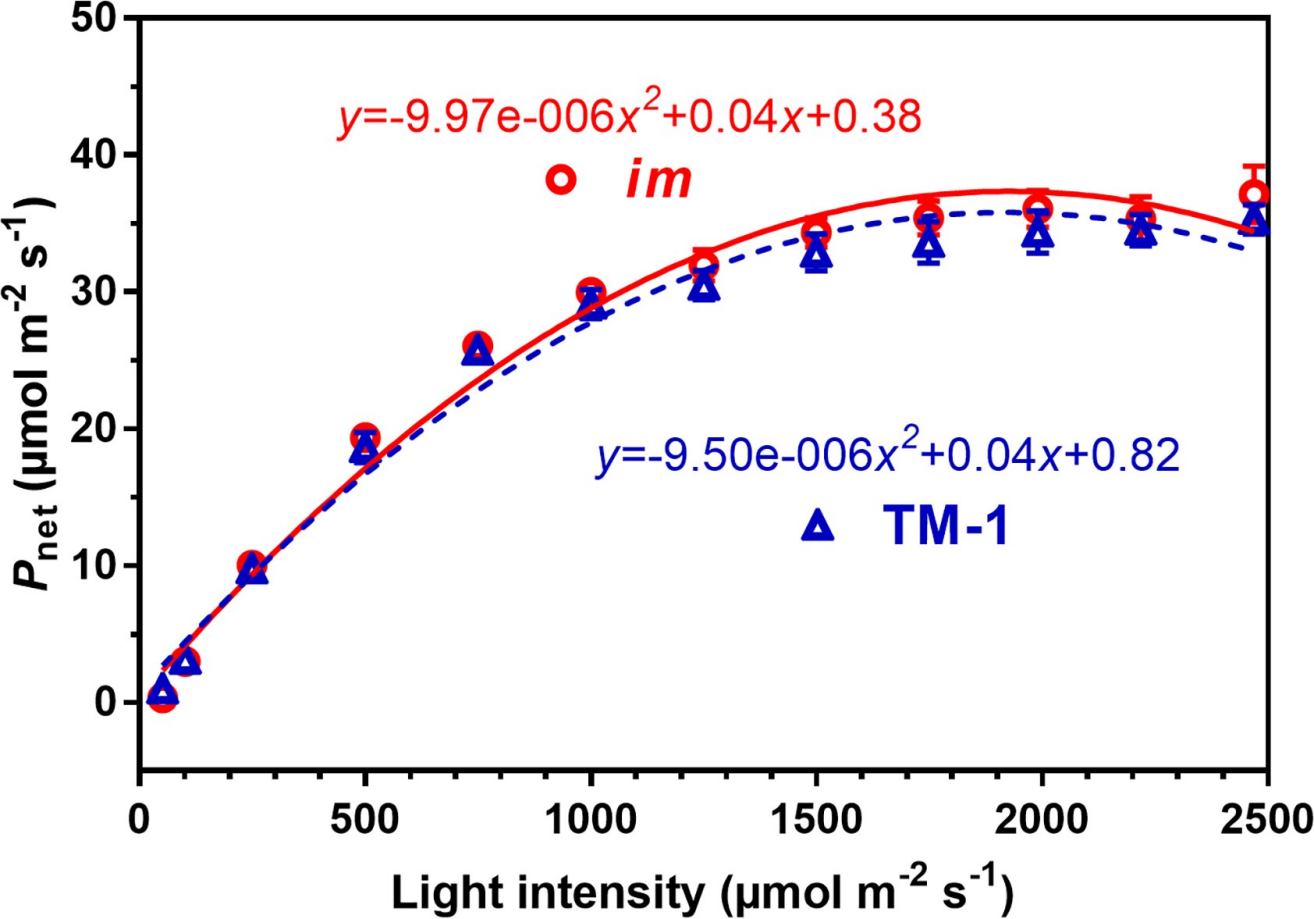
Light response curves of net photosynthesis [*P*_net_] in the field-grown *im* and TM-1 plants. Measurements were taken from the leaf at the 18^th^ node, top canopy, of four replicate plants in each NIL at approximately 31°C with varying light intensity. Error bar represents ± standard error of the mean.

To further verify the photosynthetic similarity between the NIL leaves, rapid photosynthetic light responses of the leaves at the 18^th^ node were compared under the PAR (photosynthetically active radiation) levels ranging from 0 to 2,500 µmol m^-2^ s^-1^ at a constant temperature (~31.0°C) as shown in Fig 5. The *P*_net_ increased with increasing PAR until it reached the light saturation around 1,750 µmol m^-2^ s^-1^ in both NIL leaves. Response of *P*_net_ to changing PAR also had a similar degree of fit of second order polynomial regression in *im* (*R*^*2*^, 0.961) and TM-1 (*R*^*2*^, 0.959) plants. The *P*_net_ curves of the NIL leaves almost overlapped for the entire PAR range and showed statistically insignificant difference (*p =* 0.063, Fig 5).

### Hypersensitivity of *im* leaves to locally applied high temperature at constant light intensity

**Fig 6:**
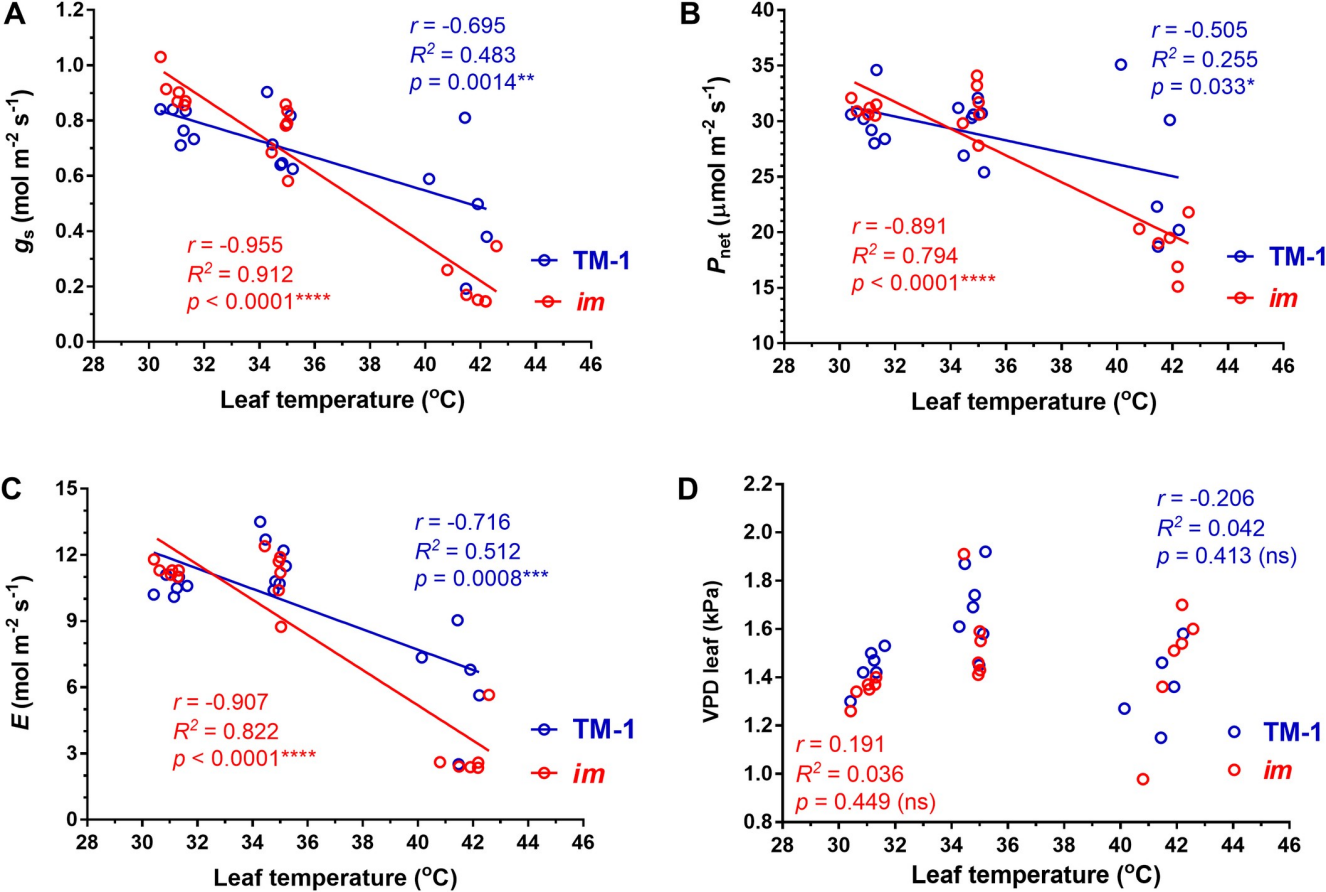
Temperature response of (A) stomatal conductance [*g*_s_], (B) net photosynthesis [*P*_net_], (C) transpiration rate [*E*], and (D) leaf vapor pressure deficit [VPD leaf] in the field-grown *im* and TM-1 plants. Eighteen measurements were taken from the leaves located at 18^th^ node of each NIL at three temperature points (30.42, 34.82, and 42.58°C, N = 6) with all other environmental variables constant.

To test if and how photosynthetic performances of the field-grown NIL leaves respond differently to high temperature, we increased the leaf temperature in the 2 cm^2^ measuring area of the leaves at the 18^th^ node from an average of 30.42°C through 34.84°C to 42.58°C using the LICOR 6400XT leaf temperature control while maintaining all other environmental variables constant. Gas exchange measurements were taken after each leaf temperature and photosynthesis at that temperature stabilized. As the leaf temperature increased from 30.42°C to 42.58°C, stomatal conductance (*g*_s_, Fig 6A), net photosynthesis (*P*_net_, Fig 6B) and transpiration (*E*, Fig 6C) commonly showed distinctive responses between *im* and TM-1 leaves. The *g*_s_ of the *im* leaves (Fig 6A) was more significantly and negatively correlated (*r* = −0.955 and *R*^*2*^ = 0.912) with the increasing leaf temperature than the TM-1 (*r* = −0.695 and *R*^*2*^ = 0.483). The *P*_net_ and *E* (Fig 6B and 6C) also showed more negative correlation to the leaf temperature in *im* leaves compared with the TM-1. However, leaf vapor pressure deficit (VPD leaf, Fig 6D) showed no significant correlation to the transient increase in leaf temperature within the measuring sample.

**Fig 7:**
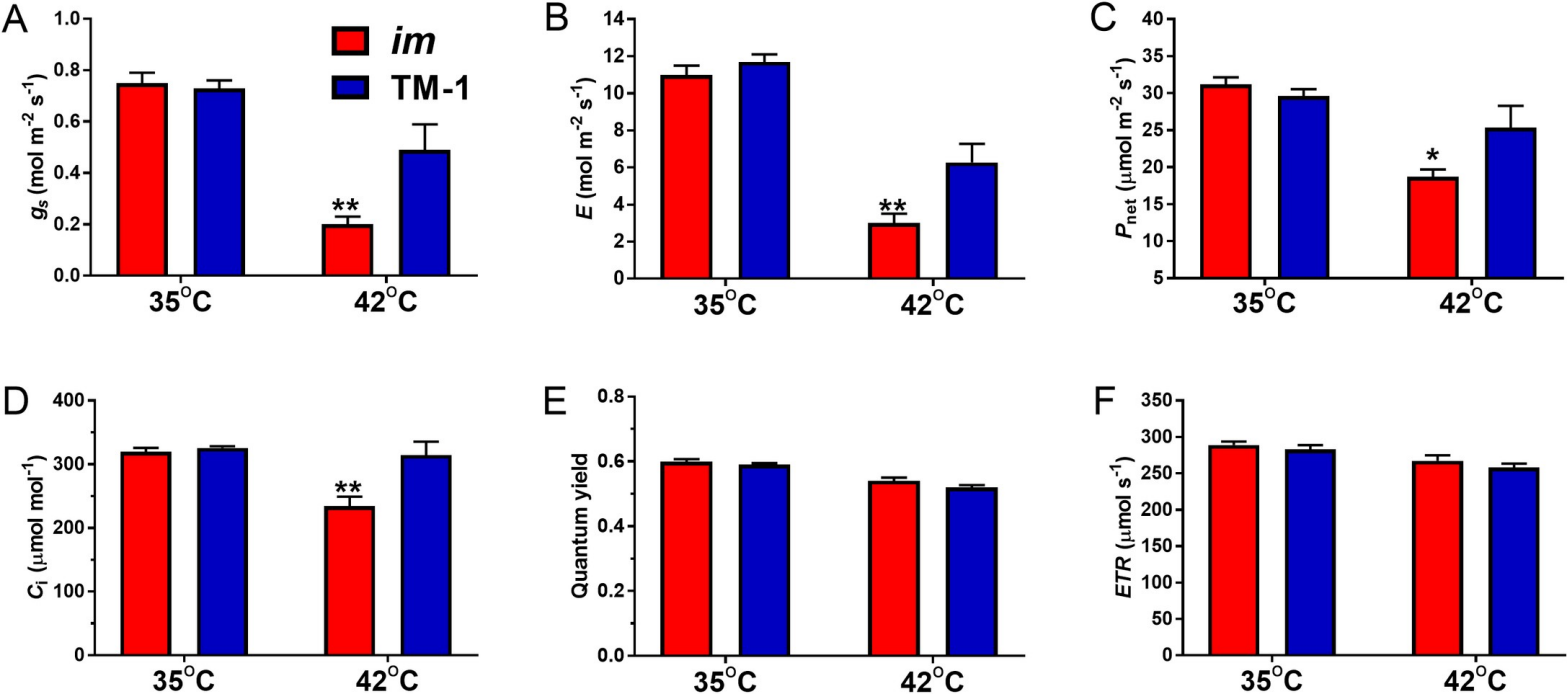
Variables of gas exchange and photosystem II function of the NIL leaves at the top canopy under locally applied high temperature treatment. A. *g*_s_, stomatal conductance; B. *E*, transpiration rate; C. *P*_net_, net photosynthesis; D, *C*_i_, intercellular CO_2_ concentration; E. Quantum yield; F. *ETR*, electron transport rate. * and ** above the bar indicate statistical significance between *im* and TM-1 at a given leaf temperature (*p* < 0.05 and 0.01), N = 6. Error bar represents standard error of the mean.

We also measured the light-adapted photosystem II activity of the NIL leaves in addition to gas exchange variables using the same LICOR 6400XT chamber equipped with the fluorometer (Fig 7). All gas exchange characteristics (Fig 7A–7D) and the light-dependent variables such as quantum yield and electron transfer rate (*ETR*) shown in Fig 7E and 7F commonly declined as the leaf temperature increased from 35 to 42°C in the NILs. None of the six variables showed a significant difference between the NILs at 35°C (Fig 7). However, gas exchange characteristics of the *im* leaves were significantly lower than the corresponding TM-1 leaves at 42°C (Fig 7A–7D). The *g*_s_ dropped much more severely in the *im* leaves (73.3%) compared with the TM-1 leaves (38.7%) as the leaf temperature increased from 35 to 42°C (Fig 7A). These data confirm the hypersensitivity of stomatal activity to high temperature in the *im* compared with the TM-1 leaves. Similarly, the reductions of the *E*, (72.7%), *P*_net_ (40.1%), and *C*_i_ (26.8%) in *im* leaves were more severe than the reductions of the respective variables (46.4%, 14.5%, and 3.5%) in the TM-1 leaves as the leaf temperature increased from 35 to 42°C (Fig 7B–7D). In contrast, neither quantum yield (Fig 7E) nor electron transport rate (Fig 7F) showed a significant difference between the NILs that were treated with 35 or 42°C.

### Comparative transcriptomic analyses of the NIL leaves

**Fig 8:**
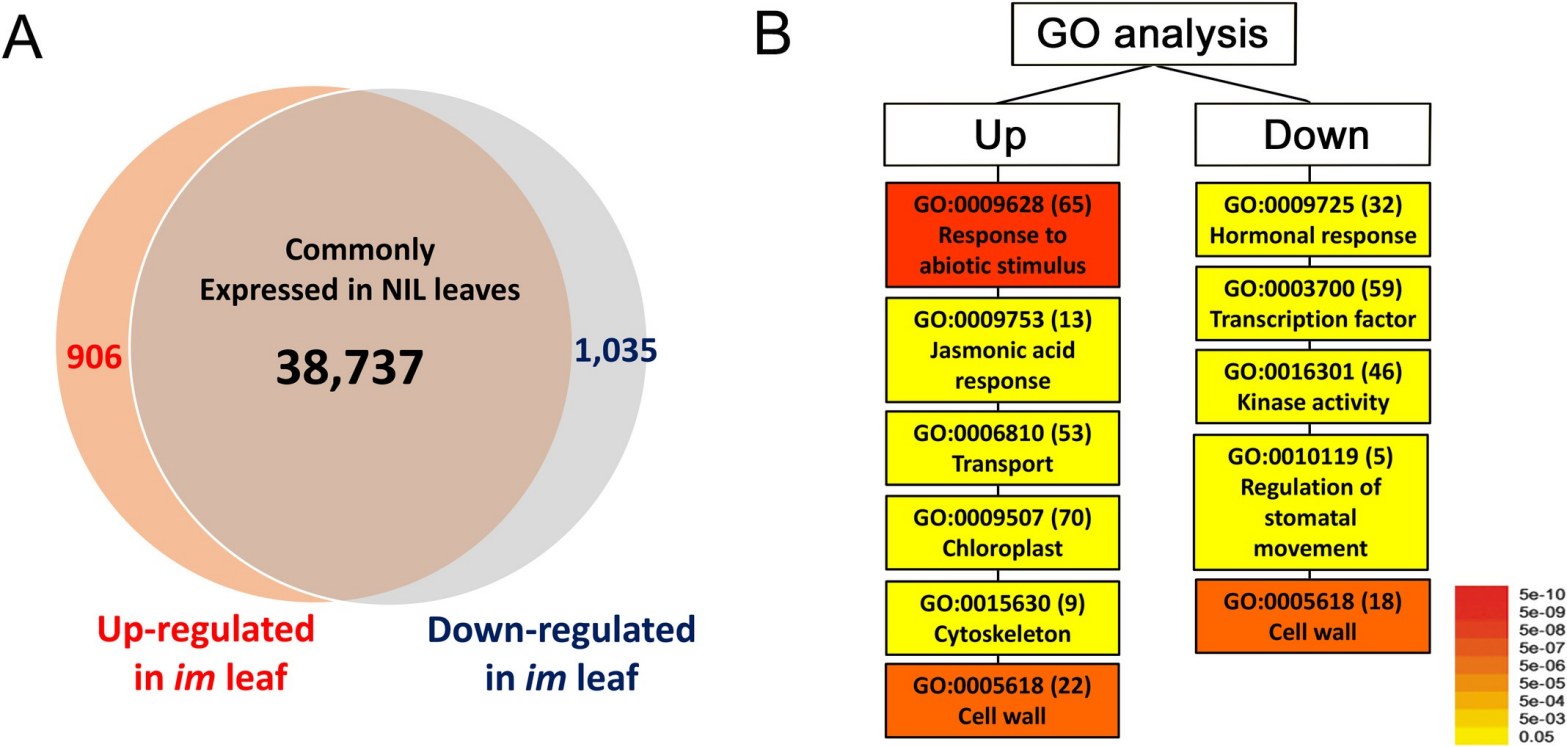
Summary of leaf RNA-seq analysis comparing the NIL plants heat-stressed at 35°C. A. Venn diagrams illustrating the up- and down-regulated genes in the *im* leaves. B. GO analysis of the up- and down-regulated genes in *im* leaves. The number of differentially expressed genes is shown in parenthesis and the color represents *p* values.

To investigate the molecular basis of hypersensitivity of *im* leaves to heat stress, comparative transcriptomic analyses between the NIL leaves were performed (Fig 8). The NIL plants were well-watered during the period of the high temperature treatment in the incubator and none of the NIL leaves wilted after the treatment. Total RNAs were extracted from the heat-treated NIL leaves and used for comparing transcript abundance by an RNA-seq. Number of the raw reads per library obtained by paired-end Illumina sequencing ranged from 47,600,508 to 55,363,510. Of the raw reads, 84.6 to 88.1% of reads per library were mapped to annotated protein coding genes in the GhHAU reference genome [38] along with the organelles’ DNA sequences (DQ345959 and JX065074). Of the 40,678 expressed (>1 RPKM) genes, 1,941 were DEGs from the *im* leaves resulting in more than a 2-fold difference in transcript abundance compared to TM-1 (Fig 8A and S1 Table). Singular Enrichment Analysis (SEA) [41] of the 906 up-regulated DEGs in the *im* leaves identified five major GO categories involved in chloroplast (70 genes), response to abiotic stimulus (65 genes), transport (53 genes), cell wall (22 genes), response to jasmonic acid (13 genes), and microtubule cytoskeleton (9 genes) as shown in Fig 8B and Table 2. In contrast, another enrichment analysis of the down-regulated 1,035 DEGs in the *im* leaves found five GO classes including transcription factors (59 genes), kinase activity (46 genes), hormonal responses (32 genes), cell wall (18 genes) and regulation of stomatal movement (5 genes) as shown in Fig 8B and Table 2. Many genes encoded in the mitochondria genome involved in the respiration pathway responsible for the *im* phenotype were down-regulated (S2 Table). Multiple photosynthesis genes encoded in the chloroplast genome were down-regulated (S3 Table) in contrast to the photosynthesis genes encoded in the nuclear genome (Chlorophyll A/B binding protein 1, Light-harvesting chlorophyll-protein complex I subunit A4, and Photosystem II light harvesting complex gene 2.1) which were up-regulated (Table 2).

**Table 2:** Annotation of differentially expressed genes and their relative degree of up- or down-regulation in the *im* leaves with reference to TM-1 from the plants incubated at 35°C for 3 days.

Cotton gene ID	*Arabidopsis* best hit*	Annotation	Transcript fold (*im*/TM-1)	Adjusted *p*-value
**Response to temperature stimulus**				
Ghir_A12G021090	AT4G25200	Mitochondrion small heat shock protein 23.6	3.93	0.05
Ghir_A11G010900	AT2G46790	Pseudo-response regulator 9	3.42	0.02
Ghir_D05G014020	AT1G54050	HSP20-like chaperones superfamily protein	3.36	0.06
Ghir_A05G017830	AT4G04020	Fibrillin	3.01	0.05
Ghir_A02G000900	AT3G59770	SacI homology protein / WW domain-containing protein	3.00	0.05
Ghir_A11G021150	AT2G22240	Myo-inositol-1-phosphate synthase 2	2.45	0.02
Ghir_A13G016230	AT2G38470	WRKY DNA-binding protein 33	2.40	0.03
Ghir_D01G001010	AT1G17870	Ethylene-dependent gravitropism-deficient and yellow-green-like 3	2.36	0.00
Ghir_A10G024160	AT5G05580	Fatty acid desaturase 8	2.28	0.02
Ghir_A01G009870	AT4G24190	Chaperone protein htpG family protein	2.25	0.04
Ghir_D01G009850	AT1G42970	Glyceraldehyde-3-phosphate dehydrogenase B subunit	2.16	0.02
Ghir_D11G001970	AT5G42020	Heat shock protein 70 (Hsp 70) family protein	2.06	0.00
Ghir_D05G012050	AT1G15950	Cinnamoyl coA reductase 1	2.03	0.00
Ghir_A10G021670	AT5G04530	3-ketoacyl-CoA synthase 19	2.02	0.00
Ghir_A12G016320	AT1G24620	EF hand calcium-binding protein family	2.00	0.06
Ghir_A04G005060	AT5G65060	K-box region and MADS-box transcription factor	2.00	0.09
**Chloroplast**				
Ghir_A05G015460	AT1G29930	Chlorophyll A/B binding protein 1	6.12	0.01
Ghir_D07G007230	AT3G47470	Light-harvesting chlorophyll-protein complex I subunit A4	4.33	0.00
Ghir_D07G018080	AT2G05100	Photosystem II light harvesting complex gene 2.1	4.31	0.00
Ghir_D10G004420	AT2G34430	Light-harvesting chlorophyll-protein complex II subunit B1	4.05	0.02
Ghir_A08G007170	AT1G03130	Photosystem I subunit D-2	3.42	0.00
Ghir_A11G015580	AT1G61520	Photosystem I light harvesting complex gene 3	3.26	0.13
Ghir_D05G025270	AT4G10340	Light harvesting complex of photosystem II 5	2.92	0.01
Ghir_A05G017330	AT4G05180	Photosystem II subunit Q-2	2.83	0.01
Ghir_D07G011920	AT1G08380	Photosystem I subunit O	2.23	0.08
Ghir_D01G016230	AT3G61470	Photosystem I light harvesting complex gene 2	2.13	0.02
Ghir_A03G023470	AT1G30380	Photosystem I subunit K	2.02	0.00
**Stomatal movement regulation**				
Ghir_D10G001280	AT1G62400	Protein kinase superfamily protein	0.43	0.01
Ghir_A01G002910	AT4G16110	Response regulator 2	0.43	0.14
Ghir_D03G008610	AT1G64060	Respiratory burst oxidase protein F	0.50	0.09
Ghir_D11G005700	AT4G33950	Protein kinase superfamily protein	0.43	0.19
Ghir_A13G020900	AT4G26080	Protein phosphatase 2C family protein	0.37	0.08
**Transport**				
Ghir_A12G025560	AT2G18196	Heavy metal transport/detoxification superfamily protein	3.83	0.00
Ghir_D05G030100	AT2G39130	Transmembrane amino acid transporter family protein	3.58	0.00
Ghir_D09G007500	AT1G61790	Oligosaccharyl transferase complex/magnesium transporter	3.50	0.01
Ghir_A11G010210	AT5G41760	Nucleotide-sugar transporter family protein	3.00	0.05
Ghir_D11G013270	AT3G51895	Sulfate transporter 3;1	2.90	0.13
Ghir_D05G015830	AT1G06330	Heavy metal transport/detoxification superfamily protein	2.70	0.00
Ghir_D13G025320	AT5G60790	ABC transporter family protein	2.67	0.18
Ghir_D11G016570	AT1G05300	Zinc transporter 5 precursor	0.45	0.17
Ghir_D01G024110	AT1G23090	Sulfate transporter 91	0.43	0.01
Ghir_A13G001050	AT1G32450	Nitrate transporter 1.5	0.27	0.00
**Abscisic acid response**				
Ghir_D05G020680	AT4G25000	Alpha-amylase-like	0.04	0.08
Ghir_D13G021720	AT5G57050	Protein phosphatase 2C family protein	0.31	0.02
Ghir_A10G010940	AT2G33380	Caleosin-related family protein	0.33	0.03
Ghir_A13G020900	AT4G26080	Protein phosphatase 2C family protein	0.37	0.08
Ghir_D03G017970	AT5G47390	Myb-like transcription factor family protein	0.41	0.07
Ghir_D11G005700	AT4G33950	Protein kinase superfamily protein	0.43	0.19
Ghir_A03G020680	AT3G19290	ABRE binding factor 4	0.45	0.00
Ghir_A09G000790	AT3G59030	MATE efflux family protein	0.45	0.01
Ghir_D05G020770	AT1G45249	Abscisic acid responsive elements-binding factor 2	0.48	0.06
Ghir_A07G023330	AT3G28580	P-loop containing nucleoside triphosphate hydrolases	0.49	0.18
Ghir_D13G017770	AT3G28910	Myb domain protein 30	0.49	0.00
Ghir_D11G002130	AT1G12420	ACT domain repeat 8	0.50	0.20
Ghir_A11G034130	AT5G16770	Myb domain protein 9	0.50	0.08
Ghir_A03G020430	AT4G37260	Myb domain protein 73	0.50	0.01
Ghir_D03G008610	AT1G64060	Respiratory burst oxidase protein F	0.50	0.09

* The sequences of the cotton DEGs were compared with *Arabidopsis* sequences (TAIR 10) and annotated based on the functions of the *Arabidopsis* genes that were the best hit by BLAST search.

## Discussion

### Environmental stress and *im* phenotype

Grown under normal field conditions, height and other non-fiber phenotypes of *im* plants appear to be similar to TM-1 (Fig 1A and 1B). In contrast, there were visible morphological differences in the boll between the NILs. The bolls were less fluffy in *im* mutant relative to TM-1 (Fig 1C and 1D). Kohel and his colleagues initially pointed out in 1990 that the *im* phenotype of non-fluffy cotton bolls is similar to the tight lock bolls that were produced in normal cotton plants when grown under severe stress conditions such as drought, cold temperature, or pathogens [11]. The causative mutation of the *im* mutant was identified and verified as a 22-bp deletion in a pentatricopeptide repeat gene, *imPPR* (Ghir_A03G006650) [24, 25]. The PPR proteins mainly play a role in RNA metabolism in organelles [48–50]. Recent biochemical and genetic studies with model plants have revealed that the PPR protein forms a complex ‘editosome’ with other proteins (non-PPR factors) for editing RNAs in chloroplast and mitochondria [51]. The PPR factors provide the RNA-binding specificity while the non-PPR factors including RNA editing factor interacting proteins (RIPs)/multiple organellar RNA editing factors (MORFs), organelle RNA recognition motif (ORRM) proteins, organelle zinc-finger (OZ) proteins, and protoporphyrinogen oxidase 1 (PPO1) play major roles in editing processes in organelles [49]. Interestingly, heat shock proteins (HSPs) involved in stress responses or protein folding can interact with both PPR and a non-PPR factors of the editosome [48, 52]. Our comparative transcriptomic study showed that the HSPs were differentially expressed between NIL leaves (Table 2). These differential expression patterns were also previously observed between developing NIL fibers [13]. Since the cotton fields of this study were irrigated and managed with recommended cultural practices, the *im* plants had little chance to be exposed to drought, flood, or nutrient deficiencies. However, the temperature was not controlled. For irrigated upland cotton, 28 ± 3°C is considered the optimum temperature [53]. High temperature stress (>34°C) induces flower sterility and reduces fiber production and quality [46, 47] whereas low temperature stress (<20~23°C) reduces metabolic activity [44, 45]. Although the threshold of high temperatures damaging individual reproductive organs might be variable among the published reports [46, 47, 54, 55], high temperatures over 35°C have consistently shown negative impacts on net photosynthesis (reviewed in [56]). In this study, the field-grown NIL plants were exposed to temperatures over 35°C every afternoon during the active fiber developmental stage (S1 and S3 Figs). Therefore, we selected the three temperatures including an optimum temperature range (31°C) and two high temperatures (35 and 42°C) for comparing the net photosynthesis and stomatal conductance of the two NILs differing in fiber wall thickness and expressions of stress responsive genes (Figs 6 and 7).

### Identification of *im* phenotype in non-fiber tissues

The *im* phenotype had been considered specific to fiber tissue since Kohel and his colleagues generated the NIL plants differing in their MIC values that were correlated with the cell wall area of cross-sectioned fibers [11, 14]. However, little phenotypic variations had been reported from the non-fiber tissues between the NILs as shown in Fig 1. Thus, these NIL plants have been used as a comparative model for investigating molecular mechanisms responsible for fiber maturity and/or SCW biosynthesis that are major determinants of the commercial value of cotton fiber and essential for the structure and biology of all land plants [21, 22]. A recent study has shown that the reduction of the functional imPPR in the *im* mutant decreased the efficiency of splicing of *nad7* and eventually reduced mitochondrial complex I activity [25]. Similar to the cotton *im* mutant, *Arabidopsis* null mutants (*slo2*, *slo3*, *slo4* and *mtl1*) which have mutations in four *PPR* genes (AT2G13600, AT3G61360, AT4G38010 and AT5G64320, respectively) show deficiency in complex I of their mitochondria [57–60]. These *Arabidopsis* mutants showed a slow growth phenotype including retarded leaf emergence, restricted root growth, and late flowering. However, unlike the *Arabidopsis PPR* null mutants in which the *PPR* was completely knocked out in its diploid genome [57–60], the levels of the *imPPR* transcripts were only partially reduced in the upland cotton *im* mutant compared with the wild type TM-1 [25]. The upland cotton (*G*. *hirsutum*) consists of an allotetraploid genome with two homeologous *imPPR* genes that share a high sequence similarity (97%). Although a frameshift mutation in one homeologous gene (Ghir_A03G006650) located in the A subgenome generated the *im* mutant, the other homeologous gene (Ghir_D03G012190) in the D subgenome is functional [24, 25]. Thus, the difference in the severity of the mutant phenotypes between the *Arabidopsis PPR* null mutants [57–60] and the upland cotton *im* mutant [24, 25] is likely due to the PPR dosage-dependent effect on mitochondrial *nad7* splicing. The *imPPR* gene is also constitutively expressed in cotton tissues. Thus, the reduction of functional imPPR protein is expected to be present in the non-fiber tissues as well as fibers of the *im* mutant [24, 25]. In this study, we found that both LMA and total leaf biomass were significantly reduced in the *im* plants compared with the TM-1 (Table 1 and Fig 3). The lower LMA value of *im* compared with TM-1, in particular, suggested that the *im* leaves were thinner than TM-1 leaves. The reduction in LMA (9.4%) was relatively less than the reduction in MIC (24.2~41.3%). The LMA shows robust relationships with the status of biomass, growth, water availability, or photosynthetic efficiency [61–63]. Thus, the data on LMA and biomass accumulation in leaf provided clear evidence that the *im* phenotype was present in the non-fiber tissues as well. However, the lack of visibly pronounced difference in LMA and leaf biomass and their low commercially motivated scrutiny may be the reasons why the leaf phenotypic differences between the NILs remained unreported so far.

### Differential regulations of stomatal conductance in the *im* mutant

Photosynthetic acclimation is a well-known plant response to the environmental changes [26–29]. Photosystem function determined by leaf chlorophyll fluorescence variables is also used to evaluate the degree of environmental stress that plants experience [28]. Among the photosynthetic parameters, maximum PSII efficiency (*F*_v_/*F*_m_) is most frequently used as an early indicator of plant stresses [30, 64]. The field-grown NIL leaves at the optimum temperature range (~31°C) commonly showed high *F*_v_/*F*_m_ (0.82–0.84) indicating little or no stress (Fig 4A) [64, 65] during our measurements taken in the mornings. Under these non-stressed field conditions, the photosynthetic similarity between NILs maintained irrespective of the light intensity (Figs 4 and 5). In contrast, the *im* leaves with the non-stress conditions achieved a higher stomatal conductance (*g*_s_) than the TM-1 leaves (Fig 4E). Stomatal conductance represents the rate of water vapor exit through the stomatal pores of a leaf, and is correlated with the size of stomatal aperture regulated by the guard cells [27]. Thus, the greater *g*_s_ in *im* suggests that the stomata of the *im* leaves opened more widely for maintaining the same degree of acquisition and assimilation of carbon as TM-1 (Figs 4 and 5). Stomata play a critical role in regulating gas exchange between the plant and its surroundings as plants respond to environmental changes and optimize photosynthesis [66, 67]. In addition, thin *im* leaves with low LMA can be heated by air temperature more easily than thick TM-1 leaves with high LMA [68]. Thus, given the similar *C*_i_ and *P*_net_ between the NILs under unstressed conditions, the wider stomatal pores of the thin *im* leaves may be required for cooling down the leaf temperature even in the morning hours in summer.

To determine the effect of high temperature stress on the stomatal conductance of the *im* leaves, high temperature (>34°C) was applied locally to the photosynthetic measuring sample of the leaves for a short term in the field-grown plants (Figs 6 and 7) and a long term to the potted whole plants in the growth chamber (Fig 8) to determine if the NILs show differential heat responses to transcriptomic profiles. As the leaf temperature of the field-grown NIL plants was raised from 30 to 35 and finally to 42°C with all other environmental variables maintained constant using the LI 6400XT photosynthesis system, the gas exchange variables including *g*_s_ and *P*_net_ were commonly reduced (Figs 6 and 7). Local high temperature treatment of the leaf photosynthetic measuring sample caused the most distinct reductions in the *g*_s_ and *E* (Fig 7) of the *im* plants suggesting that their stomata closed disproportionately and reduced gas exchange markedly compared with TM-1. The linear regression lines of the *g*_s_ against increasing temperature between the NIL leaves intersected at approximately 35°C (Fig 6A) suggesting that the temperature above 35°C caused more stomatal closure and reduced gas exchange including photosynthesis in *im* compared with the TM-1 leaves (Fig 6A and 6B). Whole plant heat stress, as opposed to the local and transient heat treatment, can be generated with a sudden increase in ambient maximum temperature by 5–7°C and retaining the whole plants at that increased temperature for a few days [69]. We subjected the potted whole plants, previously acclimated to 28 ± 3°C, to 35°C and held them at that temperature for 3 days to study the heat stress response (Fig 8). Reduced stomatal conductance toward minimizing the water loss with an accompanying drop in photosynthesis is a well-known heat response [31]. However, stomatal opening, therefore *g*_s_, is influenced by a variety of signals stemming from both external (atmospheric or soil) and internal (hormone, stress and plant systems such as root) factors [70–72]. Light intensity and quality, CO_2_ concentration, temperature, and the water vapor-related variables such as humidity or VPD are well-known atmospheric environmental factors affecting stomatal opening [73–76]. Guard cell response to these environmental cues is coordinated by a complex of signal transduction networks with inputs from CO_2_, ABA and Ca^2+^ [70, 71, 77, 78] as also discussed further in the next section, molecular mechanisms of temperature stress response in *im* leaves. Thus, while the current hydraulic status of the plant is an important determinant of the stomatal regulation, how the guard cells control the stomatal aperture in a specific environment is yet to be resolved [70, 79].

In our study, the differential response of the stomatal conductance and other gas exchange variables (Figs 4, 6 and 7) to the high temperature stress showed that the *im* leaves were hypersensitive to heat stress compared with the TM-1. Especially, the use of transient and locally applied high temperature to the leaf in the field likely allowed for the detection of leaf temperature effects on *g*_s_ more specifically before other factors affected the stomatal aperture. For instance, leaf VPD and *g*_s_ were not correlated during this transient temperature treatment (Figs 6D and S4). However, given that *im* is a mutant deficient in secondary wall thickening process of the fiber, it is worthwhile investigating if the xylem anatomies are different between the NILs as an explanation to how *im* plants maintain their hydraulic status needed for greater *g*_s_ than TM-1.

Although stomata open in response to increasing leaf temperature initially, further increase in leaf temperature beyond a threshold induce stomatal closure [75, 80].

The greater stomatal conductance of *im* compared to TM-1 plants under normal temperature (28±3°C, Fig 4) indicates that *im* plants sense a need to cool even at normal temperature. Therefore, increased stomatal closure resulting in reduced cooling during at least the times with highest temperature in the field (S1 and S3 Figs) may cause a greater metabolic stress/impairment to *im* than TM-1. The immature fiber and thinner leaves seen in *im* are likely manifestations of this impairment. As the temperature cools again in the night and continues to be optimum through the morning the gas exchange in *im* appears to recover on a daily basis but the cumulative effects of heat units are reflected in the immature fiber and thinner leaves.

### Molecular mechanisms of temperature stress response in *im* leaves

**Fig 9:**
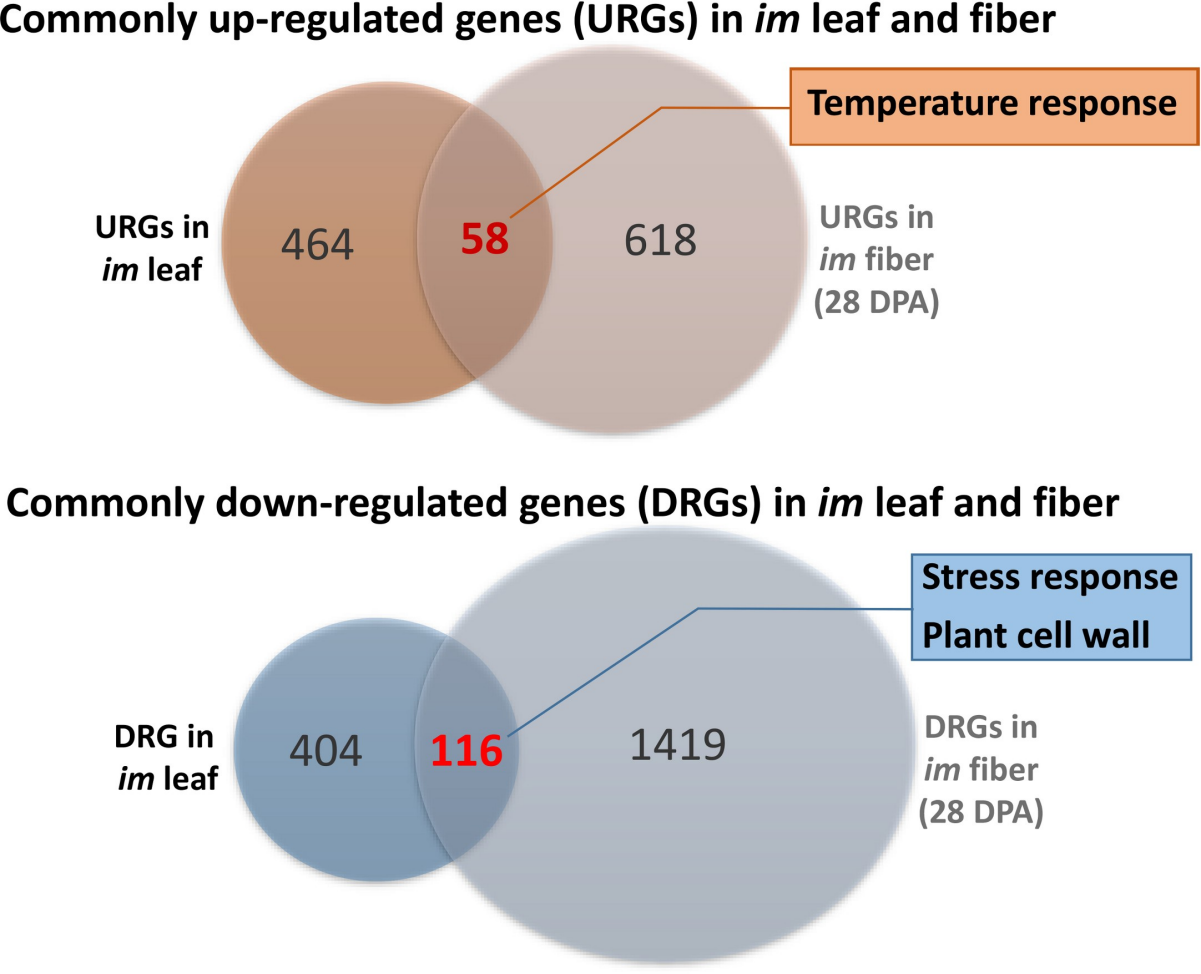
Venn diagrams showing the numbers of commonly regulated genes in leaf and fiber tissues of the *im* plant. Commonly up-regulated genes (URGs) and down-regulated genes (DRGs) were identified by comparing the DEGs determined from the NIL leaves (Fig 8) with the DEGs previously identified from developing NIL fibers at 28 DPA [24]. GO analyses identified temperature responses (GO:0009266) from the common 58 URGs and stress responses (GO:0006950) as well as the plant cell wall (GO:0009505) from the common 116 DRGs (S5 Table).

Consistent with the hypersensitivity of *im* leaves to high temperature stress (Figs 6 and 7), the genes involved in ABA responses, heat shock protein (HSP) synthesis, transport, cytoskeleton and stomatal movements were differentially expressed in the *im* leaves (Fig 8 and Tables 2 and S1) [31, 81–83]. As also discussed earlier, ABA is a stress-responsive phytohormone that is considered the first messenger for stomatal regulation [31]. Intracellular calcium ion and reactive oxygen species (ROS) are the second messengers that commonly involve in downstream of the stomatal regulatory pathways and abiotic stress responses [32]. Expansion and shrinking of guard cells needed for stomatal opening and closing are regulated by the turgor pressure driven by the influx of K^+^, anions, and sugar as well as restructuring of the cytoskeleton [31, 83]. Multiple factors including phospholipases, small molecules derived from membrane lipids, protein kinases and protein phosphatases also play regulatory roles in ABA signaling pathway and abiotic stress responses [31] thus affecting stomatal activity. The up-regulation of photosynthetic genes encoded in nuclear genome (Table 2 and Fig 8B) may have allowed the *im* plants to increase chloroplast function, but still insufficient to maintain the optimum photosynthetic performance under heat stress which closed their stomata more severely than TM-1 plants. Our current findings of differential gene expression in the *im* leaves compared with TM-1 are also consistent with the previous results showing up-regulations of numerous genes involved in abiotic stress response in developing *im* fiber compared with the corresponding TM-1 fibers [13]. Comparisons of the DEGs in *im* leaf (Fig 8) with the previously identified DEGs in developing *im* fibers [24] showed that 58 and 116 genes were commonly up- or down-regulated in both leaf and fiber of the *im* plant (Fig 9 and S4 Table). GO enrichment analyses showed that six temperature responding genes (GO:0009266) were commonly up-regulated in both tissue types (Fig 9 and S5 Table) whereas 17 stress responsive genes (GO:0006950) and six cell wall related genes (GO:0009505) were commonly down-regulated (Fig 9 and S5 Table). These results suggested that *im* phenotypes in both leaf and fiber are involved in some aspects of the control of common stress response pathways. Thus, the 22-bp deletion mutation of the *imPPR* gene (Ghir_A03G006650) responsible for the *im* phenotype likely interferes with the ability of the *im* mutant to respond to stress. Results from our physiological investigations presented earlier validate these findings from the analyses of gene expression.

Differential stomatal regulation revealed by physiological responses and transcriptomic profiles of the NIL leaves consistently showed that the *im* plants were hypersensitive to heat stress compared with TM-1 plants. Also, given that 28 ± 3°C is considered the optimum temperature for upland cotton [53], the field-grown NIL plants were often exposed to high temperatures between 34 and 36°C in the afternoon (S3 Fig) during the two calendar months of the active fiber development (July and August). Thus, the patterns of differential gene expression seen in the *im* plants in this study appear to collectively aid in their response to high temperature stress compared with TM-1.

### Hypersensitivity of the *im* mutant to high temperature stress via a coordination between mitochondria and chloroplasts

The reduction of the functional imPPR decreases the splicing capacity of *nad7* and causes the aberrant *im* fiber phenotype by reducing mitochondrial complex I activity [24, 25]. In the *im* leaf tissues of plant, an alternative oxidase (AOX, Ghir_D02G020520) was significantly up-regulated (1.9 fold). Under stress conditions in the *im* mutant, AOX was suggested to prevent productions of excess reactive oxygen species (ROS) by bypassing electron transfer from the mitochondrial respiratory pathway [13]. Up-regulations of AOX in response to application of antimycine inhibit electron transports in chloroplast in addition to mitochondria [84, 85]. AOX also plays an important role in maintaining photosynthetic performance under abiotic stress [86, 87], and acts as retrograde signaling that regulates nuclear gene expression in response to functional changes in the organelles, mitochondria and chloroplast [88, 89]. The retrograde signaling to the nucleus from mitochondrial and chloroplasts are linked and tightly coordinated to balance the activities of the two energy converting organelles with the nuclear activity of a plant cell [88–90]. The photosynthetic performance of *Arabidopsis PPR* null mutants (*slo2*, *slo3*, *slo4* and *mtl1*) which have deficiencies in function of complex I of their mitochondria has not been directly tested due to the dramatically stunted size of their leaves [57–60]. However, the *slo2* growth retardation phenotypes are substantially rectified when photosynthetic conditions including light dosage or CO_2_ concentration were enhanced [59].

Interactions between mitochondria and chloroplasts have been studied from a tobacco (*Nicotiana sylvestris*) cytoplasmic male sterile (CMS) mutant II that lacks the *nad7* subunit of the mitochondria complex I and shows developmental retardations and partial male sterility [91–93]. Comparative photosynthetic analyses have shown that the stomatal conductance, transpiration rate, and photosynthetic performance of the tobacco CMS II were lower than the wild type tobacco [94]. Functional mitochondrial complex I is required for optimal photosynthetic performance in normal tobacco leaves [95]. The CMS II mutant lacking the activity of mitochondrial complex I reduced leaf stomatal and hydraulic conductance under drought stress [96]. Interestingly, those photosynthetic responses of the CMS II mutant under stress are almost identical to those of the cotton *im* mutant under stress in this study. Under high temperature stress, the *im* leaves reduced stomatal conductance and transpiration rate, and eventually deceased the photosynthetic performance (Figs 6 and 7) compared with the wild type NIL. In addition, organellar gene expression of both mitochondria and chloroplast were down-regulated (S2 and S3 Tables). The organellar gene expression in higher plants also changed during acclimation and tolerance responses under environmental stress, presumably via retrograde signaling [90].

## Conclusion

The *im* phenotype, although so far reported in the fiber and seed, was observed in the leaf biomass accumulation and mass per unit area. Differential regulations of stomatal activity were also found between the NIL leaves. Under non-stressed conditions, *im* plants withstood the disadvantages of the thin leaves and maintain the same photosynthetic performance as TM-1 with greater stomatal conductance. However, under high temperature, *im* leaves reduced photosynthesis by closing stomata disproportionately more than TM-1 thus verifying the field observation that the *im* phenotype may be associated with heat stress. Cotton fiber is composed of nearly pure cellulose [3, 4], a polymer of glucose, which is produced with the carbon derived from photosynthesis. Thus, the high temperature stress that reduces photosynthesis more severely in the *im* plants may also contribute to their reduced fiber maturity compared with TM-1. These findings from the two NILs provide insight into how the environmental stress may involve in amplifying the *im* phenotype by meddling with the gas exchange physiology. The leaf growth and photosynthetic characteristics in the *im* plants compared with TM-1 reported here will potentially provide valuable clues for selecting the right genetic material and the growing environment that will help produce a desired fiber maturity.

## Supporting information

S1 Fig Heat map representing weekly average maximum temperatures during active fiber development in three field seasons. (PDF)

S2 Fig Heat map representing weekly average minimum temperatures during active fiber development in three field seasons. (PDF)

S3 Fig A daily temperature record during fiber development in a cotton season. (PDF)

S4 Fig Comparisons of leaf vapor pressure deficit [VPD leaf] with (A) stomatal conductance [gs] and (B) net photosynthesis [Pnet] from the field-grown im and TM-1 leaves.
(PDF)

S1 Table Annotation of genes that were differentially expressed in *im* and TM-1 leaves incubated at 35°C air temperature for 3 days. The sequences of the cotton DEGs were compared with *Arabidopsis* sequences (TAIR 10) and annotated based on the functions of the *Arabidopsis* genes that were the best hit by BLAST search.
(XLSX)

S2 Table List of *im* mitochondria genes showing more than 2-fold reduction as compared with TM-1 mitochondria genes when the NIL leaves incubated at 35°C air temperature for 3 days. Cotton mitochondria genes were compared with the *G*. *hirsutum* mitochondrial genomes (JX065074).
(XLSX)

S3 Table List of *im* chloroplast genes showing more than 2-fold reduction as compared with TM-1 chloroplast genes when the NIL leaves were incubated at 35°C air temperature for 3 days. Cotton chloroplast genes were compared with *G*. *hirsutum* plastid genomes (DQ345959).
(XLSX)

S4 Table Differentially expressed genes in both leaf and fiber of *im* plants over those of TM-1 plants. The sequences of the cotton DEGs were compared with *Arabidopsis* sequences (TAIR 10) and annotated based on the functions of the *Arabidopsis* genes that were the best hit by BLAST search.
(XLSX)

S5 Table Gene Ontology enrichment analyses of commonly up-regulated genes (GO:0009266: Response to temperature stimulus) and commonly down-regulated genes (GO:0006950: Response to stress; GO:0009505: Plant-type cell wall) in both leaf and fiber of *im* plants over those of TM-1 plants. (XLSX)

S1 Graphic abstract (TIF)
