# Supplementary material for: Evidence for thermosensitivity of the cotton (Gossypium hirsutum L.) immature fiber (im) mutant via hypersensitive stomatal activity
Source: PLoS One. 2021 Dec 13;16(12):e0259562. doi: 10.1371/journal.pone.0259562 (PMC8668099; doi:10.1371/journal.pone.0259562)
Supplement: S1 Fig — (PDF) [file pone.0259562.s001.pdf]

S1 Figure

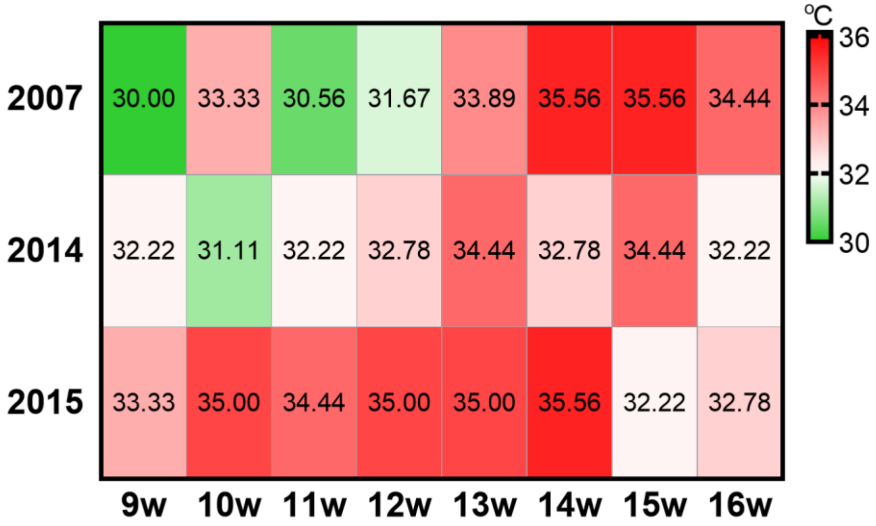

S1 Fig. Heat map representing weekly average maximum temperatures during active fiber development (9th to 16th week of the growing seasons) in three field seasons.
