# Supplementary material for: Evidence for thermosensitivity of the cotton (Gossypium hirsutum L.) immature fiber (im) mutant via hypersensitive stomatal activity
Source: PLoS One. 2021 Dec 13;16(12):e0259562. doi: 10.1371/journal.pone.0259562 (PMC8668099; doi:10.1371/journal.pone.0259562)
Supplement: S2 Fig — (PDF) [file pone.0259562.s002.pdf]

S2 Figure

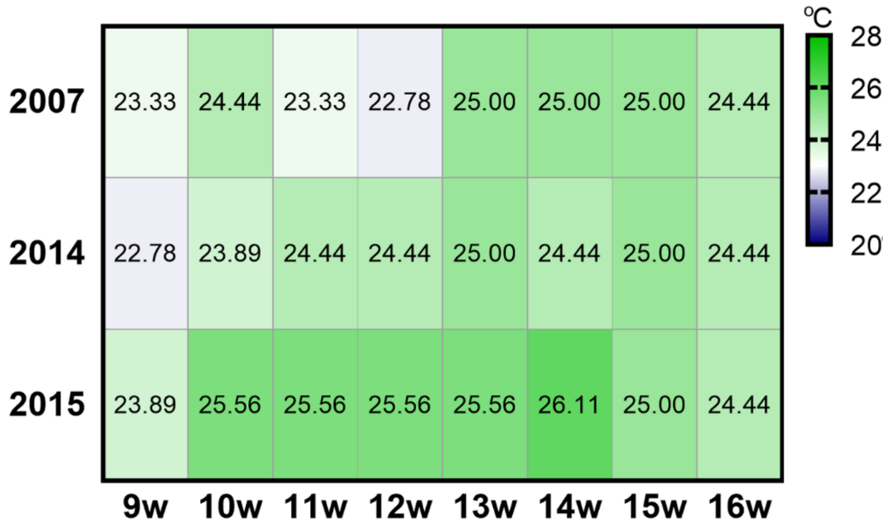

S2 Fig. Heat map representing weekly average minimum temperatures during active fiber development in three field seasons.
