# Supplementary material for: Evidence for thermosensitivity of the cotton (Gossypium hirsutum L.) immature fiber (im) mutant via hypersensitive stomatal activity
Source: PLoS One. 2021 Dec 13;16(12):e0259562. doi: 10.1371/journal.pone.0259562 (PMC8668099; doi:10.1371/journal.pone.0259562)
Supplement: S3 Fig — (PDF) [file pone.0259562.s003.pdf]

S3 Figure

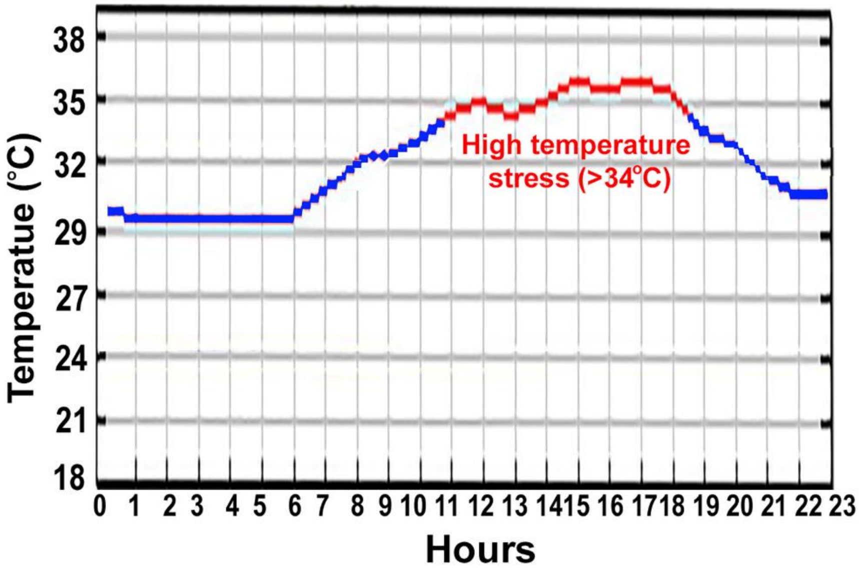

S3 Fig. A daily temperature record during fiber development in 2015 cotton season. The red line represents a period (11 A.M. to 6 P.M.) of high temperature stress (>34°C) on a representative day of July, 2015.
