# Supplementary material for: Evidence for thermosensitivity of the cotton (Gossypium hirsutum L.) immature fiber (im) mutant via hypersensitive stomatal activity
Source: PLoS One. 2021 Dec 13;16(12):e0259562. doi: 10.1371/journal.pone.0259562 (PMC8668099; doi:10.1371/journal.pone.0259562)
Supplement: S4 Fig — Comparisons of leaf vapor pressure deficit [VPD leaf] with (A) stomatal conductance [gs] and (B) net photosynthesis [Pnet] from the field-grown im and TM-1 leaves. (PDF) [file pone.0259562.s004.pdf]

S4 Figure

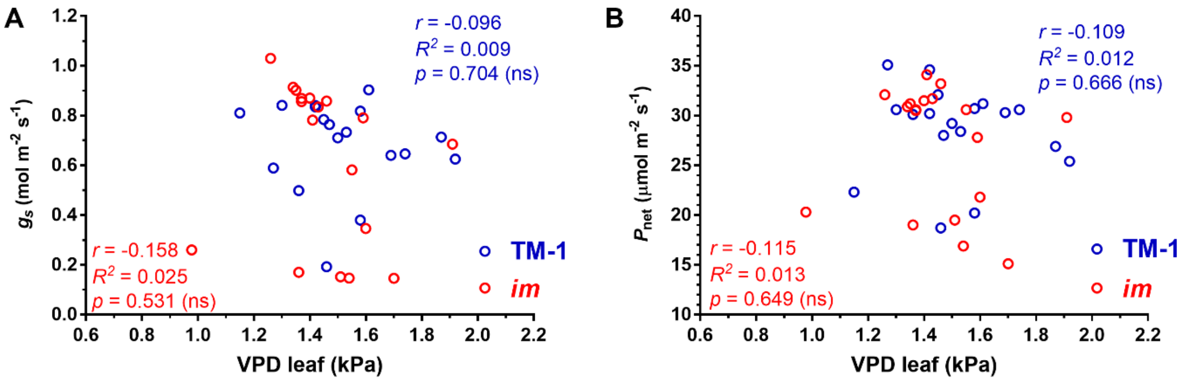

S4 Fig. Comparisons of leaf vapor pressure deficit [VPD leaf] with (A) stomatal conductance [g_s] and (B) net photosynthesis [P_{net}] from the field-grown *im* and TM-1 leaves. Six measurements were taken from the leaves located at 18th node of each NIL at 30.4, 34.8, or 42.6°C with all other environmental variables constant.
