# Supplementary figures and images for: Evidence for thermosensitivity of the cotton (Gossypium hirsutum L.) immature fiber (im) mutant via hypersensitive stomatal activity
Source: PLoS One. 2021 Dec 13;16(12):e0259562. doi: 10.1371/journal.pone.0259562 (PMC8668099; doi:10.1371/journal.pone.0259562)

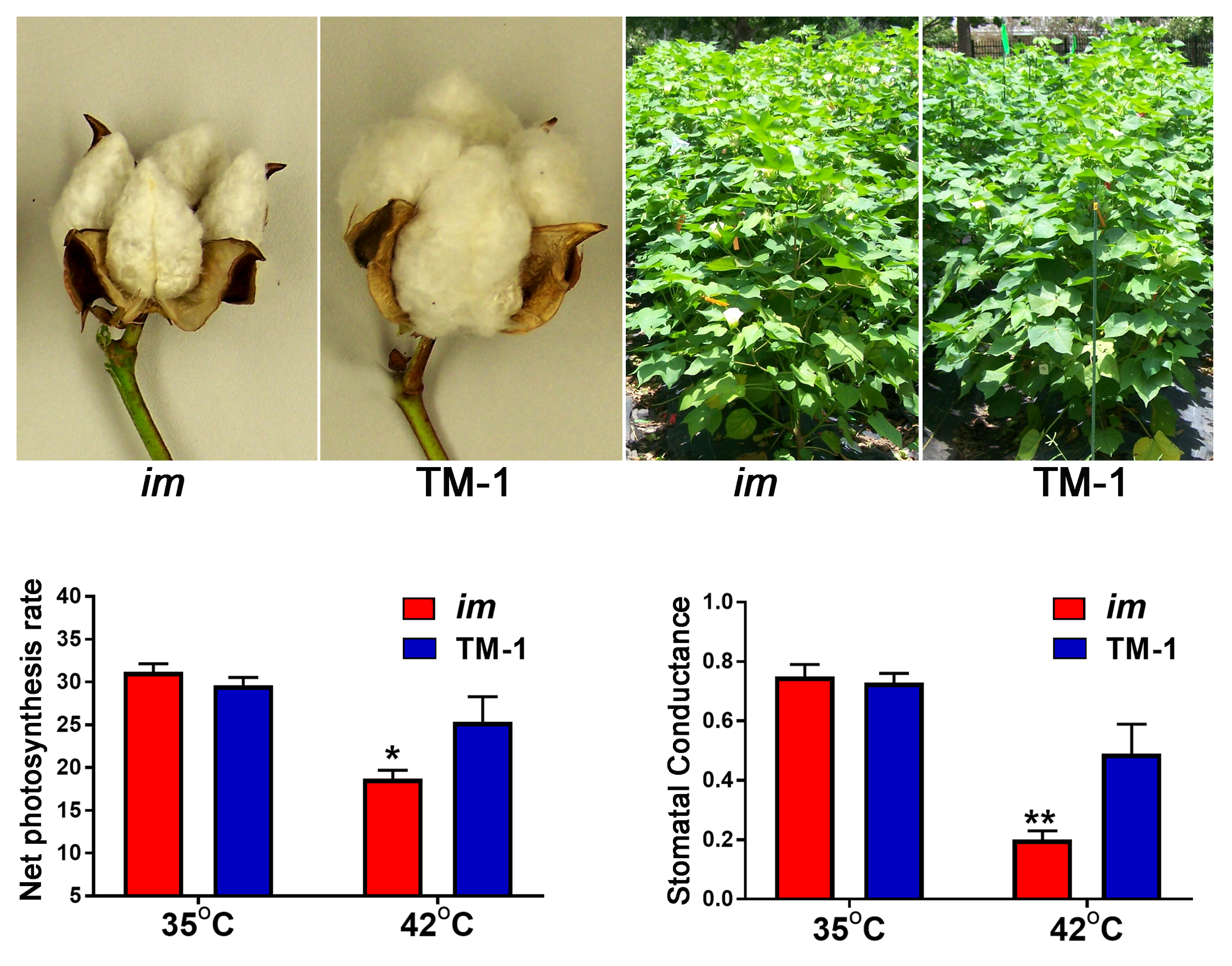

Supplement: S1 Graphic abstract — (TIF) [file pone.0259562.s010.tif]
